# New scenarios of non-violent conflict in indigenous communities: The case of REDD+ in the Colombian Amazon

**DOI:** 10.1177/27538796251330357

**Published:** 2025-04-10

**Authors:** Dominique Schmid, Carolina Castro Osorio

**Affiliations:** Department of Political and Social Sciences, Universitat Pompeu Fabra, Spain; Department of Human Geography and Spatial Planning, Utrecht University, Faculty of Geosciences, the Netherlands; Universidad de los Andes, Faculty of Economy, Colombia

**Keywords:** forest conservation, REDD+, non-violent conflict, Indigenous Peoples, Colombia, community governance, elite capture, transparency

## Abstract

Despite debates about their effectiveness, forest carbon offsets are one of the most traded credits on the voluntary carbon market. Projects like REDD+ are often implemented in the lands of Indigenous Peoples and local communities. Studies show that projects contribute to social conflict within and between participating communities, but little is known about how these conflicts unfold. This study aims to shed more light on the mechanisms that shape these conflicts through the lens of local institutional dynamics, namely community governance, elite capture, and transparency. Based on interviews conducted in 10 Indigenous communities in the Colombian Amazon involved in four REDD+ projects, we found that projects are closely associated with elite capture, with local community and community association leaders dominating the decision-making processes. This fragments trust in leaders and undermines project legitimacy. Also, the prospect of carbon payments disrupts communities and triggers conflicts over their distribution. These effects are moderated by decision-making mechanisms and are exacerbated by transparency issues. Although focused on Indigenous communities in the Colombian Amazon, our findings can inform similar contexts with autonomous community governance where REDD+ interventions are implemented.

## Introduction

The urgent need to conserve the world’s forests to mitigate climate change, conserve biodiversity, and protect the livelihood of forest-dependent communities is widely acknowledged. With forest carbon offsets comprising over 61% of the voluntary carbon market, and multilateral climate funds allocating billions to REDD+^1^ activities, the carbon storage capacity of forests has become a commodity in the pursuit of climate action (Ecosystem Marketplace, 2021; Watson et al., 2022).

REDD+ is a UNFCCC^2^ framework designed to reduce deforestation-related emissions and promote sustainable forest management in developing countries. REDD+ projects implement these goals locally by incentivizing Indigenous and local communities to adopt conservation and sustainable land-use practices. These projects also support land governance, community engagement, and capacity building. Each verified reduction or avoidance of CO_2_ emissions as a result of the project generates a tradable carbon credit, which can be sold to offset emissions. However, developing REDD+ projects and marketing credits requires technical expertise and financial resources—both of which local communities often lack. As a result, they must rely on external intermediaries^3^ to manage the process, secure funding, and market the credits, typically in exchange for a share of the profits.

REDD+ has fostered important positive outcomes because it has put a spotlight on the importance of securing local and customary land tenure rights (Larson et al., 2013). Also, REDD+ has strongly stressed the significance of addressing underlying governance challenges that contribute to deforestation and forest degradation (Williams & De Koning, 2016). When a REDD+ project is being planned or implemented, there are most likely different opinions between community members who oppose participation in the project and those who support the project based on principle or its perceived benefits (Massarella et al., 2018; Schilling, Saulich and Engwicht, 2018; SINCHI & Minambiente, 2022). Dealing with different views and opinions when new policies are being planned or implemented is natural in all kinds of governance systems, but when some views are marginalized, it is highly likely that conflicts will arise. This can destabilize communities by reproducing existing inequalities and further marginalizing already disadvantaged groups (Astut i& McGregor, 2017; Corbera, 2012; Vigil, 2018). The frustration around unmet project expectations and (perceived) unequal benefit-sharing has also been detected as a frequent driver of conflict (Alusiola et al., 2021). Further negative effects of REDD+ projects can include conflict and violence over land and resource access (Kemerink-Seyoum et al., 2018; Sikor & Cầm, 2016), forced evictions and displacement (Howson, 2018), or the negative impact of new land-use policies on local food security (Tabeau et al., 2017).

REDD+ projects, like other community-based and participatory projects, face the challenge of elite capture, where local elites monopolize the resources of the decision-making processes. Studies indicate that elite capture is more likely in environments with weak governance, poor transparency, and high inequality (Mansuri & Rao, 2004; Platteau & Abraham, 2002). Strong institutions with transparency mechanisms help mitigate these risks, enabling communities to manage financial resources and natural resources more effectively (Ostrom, 1990). However, anticipated and materialized streams of revenue can incentivize the use of corrupt practices, increasing the risk of elite capture (Brollo et al., 2013; Sala-i-Martin & Subramanian, 2013). Transparency is essential to prevent elite capture in both community-driven projects and in natural-resource management (Ostrom, 1990) as the lack of transparency enables elites to control information flow, giving them an advantage in determining project outcomes and resource distribution (Labonne & Chase, 2009).

Although there is relative consensus in the literature around the local effects of REDD+ projects, scholarship on REDD+ still needs to delve deeper into issues of governance and politics at the subnational level—and specifically into questions of territory—to find out how institutional and governance mechanisms influence this relationship (Cifuentes, 2021; Orihuela, 2018). Furthermore, it remains unclear whether, and to what extent, projects based on collective land tenure and self-governance have different implications. This is because the literature that analyzes the characteristics of the dilemmas of collective action faced by community-based REDD+ remains scarce (Saeed et al. 2017).

The goal of this study is to investigate these links and to evaluate the extent to which REDD+ projects implemented on collective territory contribute to non-violent conflicts such as internal divisions, disputes, contestation, or resistance within and between Indigenous communities. We analyze these conflicts through the lens of local institutional dynamics, namely community governance, elite capture, and transparency. Shedding more light on how non-violent conflict can erupt at the community level is important because it allows us to look more critically at the impact of REDD+ interventions and the unintended consequences of policies that aim to mitigate climate change.

To investigate these links, we collected empirical data from ten Indigenous communities of the Colombian Amazon that participated in one of four sampled REDD+ projects. Colombia is excellently suited to study this relationship because since the Colombian Constitution of 1991, collective land tenure rights are well-protected, and Indigenous Peoples have the right to jurisdiction over their territories in accordance with their laws and procedures (Colombia, 2015). This right to autonomy and self-governance includes sovereignty to control and negotiate the management of natural resources, which has largely eliminated the function of state control (Ortiz-T & Chirif, 2010). Colombia is also one of the few countries in the world—along with Brazil and Costa Rica—where carbon rights are legally tied to land ownership, including collective lands (Rights and Resources Initiative, 2021). This means that Indigenous communities can transfer carbon rights to other actors.

This article is structured as follows: in the next sections, we discuss the importance of trust in collective governance structures, the vulnerability of these systems to elite capture, and systemic issues about transparency. We then justify our case selection, give some context about our case studies, and outline our data collection method and analysis. This is followed by a discussion of our results. We close this article with concluding remarks.

## The importance of trust in collective governance structures

To analyze the mechanisms we are interested in, and to determine the relevance of our findings beyond the context of our case study, we have to set parameters about community governance. To do this, we use some of the basic concepts of social contract theory to outline how community governance can be constructed.

Classic social contract theory is based on assumptions of human behavior about what leads citizens to consider authority as being legitimate and thus also to consider the principles of a stable political order (Leonard, 2013). The theory assumes that the state is based on a social contract between the individual and authority (Leonard, 2013). In many Western societies, each citizen forms a social contract with the state, but in many rural societies of the Global South, individuals form this contract with their community, and communities as a unit form it with the state (Leonard, 2013). However, neither communities, nor how these social contracts are governed within and between communities, are homogeneous. For example, some communities have set governance structures where leaders are assigned (or elected to) a specific role within political, spiritual, and professional dimensions, while other communities form associations that govern across the communities (Katene, 2010).

Generally, these social contracts are built on trust. Trust is defined as the confidence placed in leaders to represent the community’s interests to the best of their ability, and to uphold historical and contemporary norms as well as the embedded governance systems of the community (Silva Dos Santos et al., 2025; Wolfgramm et al., 2016). Indigenous governance is collective and is built on shared cultural values, norms, and traditions. Trust is fundamental to the functioning of collective governance, because when individuals trust that others will contribute their fair share to the prosperity of the community, they are inclined to collaborate, and they are motivated to reciprocate (Kahan, 2003). In collective governance systems, trust serves as an indicator of the governance systems’ legitimacy—defined as the rightful authority to govern—within the community (Nikolakis & Nelson, 2019). The literature on collective land and natural-resource governance systems highlights the importance of social capital, trust, and the legitimacy of these social arrangements (Ostrom, 1990).

Developing a REDD+ project and marketing the resulting carbon credits requires technical knowledge of carbon standards and the carbon market. It also requires financial capital to cover the project development and monitoring costs; costs which local Indigenous or peasant communities normally do not possess. Because of this, communities have no choice but to work with an external intermediary that completes these tasks, markets the credits, and brings in the funding in return for a share of the profits from the sale of the carbon credits. As a result, the previous collective resource-governance system switches to become a collaborative system, because an external actor becomes involved in the decision-making and governance of the forest and other natural resources in the Indigenous territory. Also, in collaborative resource-governance systems, trust between the actors remains essential to sustain commitment to the partnership (Ansell & Gash, 2008).

## Local institutions’ vulnerability to elite capture

Many Indigenous organizations have long-standing issues with organizational capacity in political and administrative terms, which in the past has led to friction and internal divisions (Jiménez, 2007). However, third-party intervention in the management of natural resources, and the resulting benefits, can further weaken the social norms of the collective society and can result in negative effects such as elite capture and the prioritization of the individual over communal benefits. Elites are members of a society or a community who have disproportionate influence and power over decision-making (Post, 2008). In this study, we consider elites as community members with a leadership role at the community level (e.g., governors, vice-governors, spiritual healers and leaders, spokesperson) or at the community association level (AATI) (e.g., president, secretary). Elite capture can take on different forms, such as when elites take control of local policy and decision-making (Lund & Saito-Jensen, 2013) or when they capture collective monetary resources, which then results in the unequal distribution of benefits (Mrema, 2017).

REDD+ projects share many characteristics with community-based and participatory development projects including the emphasis on local leadership, broad participation, capacity building, or taking advantage of local resources and knowledge. The literature on development studies has long highlighted the vulnerability of community-based and participatory projects to elite capture (Mansuri & Rao, 2004; Platteau, 2004; Platteau & Abraham, 2002; Powis, 2007). It has been found that elite capture is more likely to occur in environments with weak governance, low levels of transparency, and high levels of inequality, as well as in projects with flawed capacity building and training of community facilitators (Platteau, 2004; Post, 2008). For example, Mansuri and Rao (2012), in their comprehensive review of almost 500 projects, show that weak local institutions and a lack of accountability mechanisms enable elites to dominate project design and implementation. Also, Bardhan and Mookherjee (2006) found that due to pre-existing inequalities, community elites can dominate the distribution of agricultural inputs and resources of community-based programs to their farms, leaving other smallholders marginalized. Communities with strong local institutions and effective transparency mechanisms are better equipped to manage potential negative impacts—such as the elite capture of incoming financial resources and material assistance—and are in a stronger position to manage their natural resources (Dongier et al., 2002; Mansuri & Rao, 2012; Ostrom, 1990). Hence, the quality and strength of local institutions do matter (Kaznacheev, 2017; Menaldo, 2016).

Natural resource wealth, including access to and control over forests and other ecosystems, can act as an incentive for corrupt practices such as elite capture (Brollo et al. 2013; Mrema 2017; Sala-i-Martin and Subramanian, 2013). This means that territorial control and autonomy over carbon contracts can also pose a challenge for communities because the risk of elite capture increases. Several studies of REDD+ and other forest-based interventions discovered that local leaders made decisions unilaterally either about using project funds or about making the decision to participate in a project, which fractures trust in leaders (e.g., Corbera et al., 2020; Mrema, 2017). Unilateral decision-making can also increase suspicion about the use of (anticipated) funds and can encourage community members to request that funds be directed to them and not channeled through leaders, which poses a significant threat to the functioning of local governance systems (Massarella et al., 2020). It can even provoke the removal of leaders: as Corbera et al. (2020) show in their study of a Payment for Ecosystem Services (PES) program in Chiapas, Mexico, a leader was forced to resign (but ultimately ended up staying in his position) because he made decisions unilaterally and allegedly misused funds for his benefit. While studies have found that the distribution of benefits by community leaders is at times equitable, it has also been shown that this can create tensions, because the distribution of benefits is considered subjectively, either between individual community members or when communities perceive that they have benefited less compared with other communities (Wallbott & Florian-Rivero, 2018).

## Systemic issues about transparency

Transparency is an important safeguard to prevent the capture of resources and decision-making by elites in community-driven projects and the management of natural resources, including the management of forests (Ostrom, 1990; Dongier et al., 2002). Based on data from 1,200 households within 66 communities in the Philippines, Labonne and Chase (2009) found that elites often control the flow of information, giving them an advantage in determining project outcomes and resource distribution. Information-sharing related to natural-resource governance has also been identified as a tool to avoid or to reduce conflict, while the lack of information-sharing has been identified as a driver of elite capture. For instance, in a study relating to the discovery of natural resources in Mozambique, Armand et al. (2020) found that information-sharing with citizens increased local mobilization and decreased violence, but when information was shared only with local leaders, elite capture increased.

Transparency remains a major issue for REDD+ projects as well as for the entire forest carbon sector. Transparency initiatives relating to carbon have largely focused on the monitoring, reporting, and verification of carbon emissions from a corporate perspective (e.g., the Carbon Disclosure Project (CDP) and the Partnership for Carbon Transparency (PACT)). But what remains largely unaddressed are existing transparency issues at the project level. For REDD+ projects, the specific content of the contracts between the intermediary and the communities—as well as the conditions of implementation and benefit-sharing—is almost never known by the public. Hence, the conditions of the collaborative governance regime remain largely undisclosed. While most project design documents (PDDs) are publicly available and mention contracts or agreements, contracts and agreements are rarely made public. Confidentiality is used as the justification for non-disclosure, but it is exactly this kind of transparency issue that can lay the groundwork for unequal project conditions and unequal benefit-sharing, which can prevent some intermediaries’ accountability for ethically and morally questionable business practices.

In collective governance settings, the head of a community or the leader(s) designated to handle the matter plays a crucial role in informing other leaders and their communities about current affairs because they represent the collective. For this system to function, transparency and access to information must be guaranteed at two levels: (a) between the project intermediary and the designated leader and (b) between the designated leader and other community members. Unfortunately, information-sharing at both levels is often not guaranteed (Montoya-Zumaeta et al., 2021). Project contracts are held secretively by those who signed them, and they are not even shared with all community leaders (Kemerink-Seyoum et al., 2018). Such issues disrupt the flow of information. This can be problematic because, for community members, the community leaders are the focal point of information and the first way to answer questions and manage expectations. When concerns remain unanswered, it can potentially promote mistrust or conflict because communities tend to compare their received or expected benefits with those of other communities, and this is likely to generate tension and raise suspicion. This results in a weakening of the collective governance system beyond the management of natural resources (Kemerink-Seyoum et al., 2018).

Lack of transparency is also prone to unequal benefit-sharing, thus causing grievances. Project intermediaries can use their superior knowledge of the market to set the terms of the project and to set benefit-sharing to their advantage (Corbera and Martin, 2015). A large difference between carbon and extractive goods—such as oil, timber, and gemstones—is that local communities tend to know that extractives are a highly valuable resource, while carbon is a non-materialized good that can be abstract, with a value that is difficult to grasp. Studies have shown that these knowledge asymmetries about what exactly is sold, and what it is worth, lay the foundation for uneven benefit-sharing in collaborative governance (Bryant et al., 2015; Wang & Corson, 2015). Studies of REDD+ and other forest carbon projects have repeatedly demonstrated negative community-level impacts over benefit-sharing both between and within communities. These negative impacts have resulted in widespread dissatisfaction about the projects and the conflicts, with some community members demanding greater transparency and equity in benefit-sharing (Kemerink-Seyoum et al., 2018; Massarella et al., 2018). The unequal and inadequate distribution of benefits from community-based, natural-resource management projects can also challenge the long-term sustainability of such initiatives (Suich, 2013).

## Case selection and context

To evaluate how REDD+ projects that are implemented on collective territory contribute to non-violent conflicts through their use of mechanisms of community governance, the prevention of elite capture, and transparency, we had to focus on a context with a decentralized governance structure that allowed for territorial autonomy below the subnational scale. We decided to focus on Indigenous communities in Colombia because they have territorial control over their lands and the right to self-governance, as described in the introduction to this article. However, our results are relevant to any other context that has a decentralized governance structure at the community territory level that is built on social values—for example, Bolivia.

Geographically, we limited our study to the Colombian Amazon region because it has the highest concentration of projects on Indigenous territory. Our case selection was then driven by (a) project status and (b) security. The first criterion was crucial because, to fulfill the research aim, we had to focus on communities that had been participating in a project for a while. This is because we wanted to allow sufficient time for socialization activities to take place. Socialization activities are conducted by the project intermediary and sometimes by delegated community members. The activities aim to inform communities about the projects and to answer any questions. So, we decided to focus on projects that had either been officially registered as carbon projects with a project registry or that had requested registration after completing all pre-project development phases. These selection criteria resulted in four projects for the study (Table 1): three projects in the Vaupés department and one in the Amazon department. The detailed case selection process is included in Appendix 1.

**Table 1.** Sampled projects (source: project design documents (PDDs), unless otherwise stated).

Project title	Intermediary/standard	Department	Communities and AATI involved (Associations of Indigenous Traditional Authorities)	Duration (yrs) and start date	Contract signed by
Makaro Ap+ro (Makaro)	Masbosques^4^ Cercarbono (EcoRegistry)	Vaupés	24 communities of which 8 are represented by the AATI “AATICAM” and 16 by the AATI “ASATRIBVA”	30/2018	Heads of the communities
Baka Rokarire ~IA TIR1~DITO (Baka Rokarire)	Masbosques Cercarbono (EcoRegistry)	Vaupés	21 communities of which 16 are represented by their AATI (ACAIPI) and 5 additional communities	30/2018	President of the ACAIPI for its 16 associated communities Legal representatives of the other 5 communities
REDD Project of the Indigenous Peoples of Vaupés YUTUCU and Others (YUTUCU)^5^	South Pole Group^6^ VCS	Vaupés	75 communities, which are represented by 5 AATIs (AATIAM, AATIVAM, ASATRAIYUVA, ASOUDIC, AZATIAC)	20/2016	Presidents of the 5 AATI
Proyecto de Mitigación Forestal Resguardo Indígena TICOYA (TICOYA)	South Pole Group BioCarbon	Amazon	22 communities, of which 21 are represented by the AATI ATICOYA, and one additional community.	20/2010	AATI president (at that time) (source: interviews)

## Case study context

The communities in our sampled cases are organized in several levels of governance (Figure 1). The biggest institutions are the Indigenous reserves (*resguardos*). This is the arrangement that establishes collective property rights over land, but they are not governmental units. Inside the *resguardos*, we find two levels of government: Indigenous communities under the leadership of a *capitan* or governor and the Associations of Indigenous Traditional Authorities (AATI). The AATIs are made up of several communities belonging to the same *resguardo.* Within a *resguardo*, there may be several AATIs. Most communities in our sample were associated with an AATI. Within the Indigenous reserves, the AATIs and the traditional authorities of the communities are the formal public governance entities (Torres & Verschoor, 2020). As such, the ATTI is an organizational structure through which the associated Indigenous communities are governed (Gaia Amazonas, n.d.). The existence of this level of organization does not compromise the autonomy of the community level. There is no uniform AATI structure because the associated communities define the structure and the corresponding levels of authority through participatory processes (ibid). Given this background, governance structures across the communities that participated in our interviews are not homogeneous. In some of our cases, the AATI president had the authority to sign the project contract, while in other cases this power was left to the leader of the communities (Table 1), which, as we will show, has a major impact on project legitimacy.

**Figure 1.**
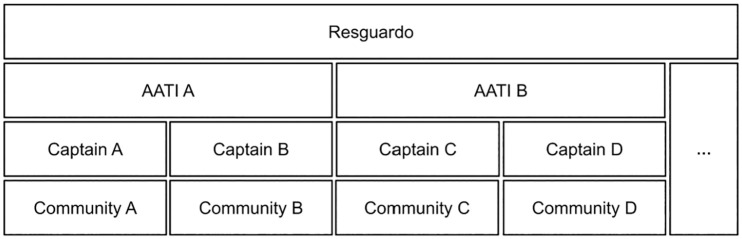
Community governance structure (simplified).

Three of our four sampled cases are located in the Vaupés department within the *Gran Resguardo del Vaupés*. This *resguardo* consists of 27 ethnic groups organized in 230 communities, which are associated with 17 AATIs (SINCHI & Minambiente, 2022). The *Gran Resguardo del Vaupés* covers a territory of 3.3 million hectares of forest (ibid). The REDD+ projects included in this study involve 120 communities and eight AATIs in total (Table 1) and span an area of more than 1.7 million hectares (AATIAM et al., 2020; Masbosques, 2021a, 2021b). The territory is classified as a highly diverse tropical rainforest with an average of 120 species per hectare and a high carbon sequestration capacity (Minambiente, 2020b). The fourth sampled case is located in the Amazonas department which consists of 13 Indigenous reserves (SINCHI, 2022). Our chosen REDD+ project is located in the Puerto Nariño Indigenous reserve, which has an area of over 142,000 hectares (AENOR, 2019). This territory is also dominated by tropical rainforest and has an estimated forest cover of over 130,000 hectares (ibid). In this project, all but one of the communities are represented by the Ticona, Cocoma, Yagua Indigenous Association (ATICOYA).

## Data collection and analysis

The empirical evidence of this study was collected in August 2022 in the form of semi-structured and unstructured interviews with 30 members from 10 communities that participated in one of the four selected projects. We distinguished between community members with a leadership role—either head of community or another leadership role within their community or at another governance level—and community members without a leadership role. We interviewed 16 leaders and 14 non-leading community members (Appendix 2). Interviewees were selected based on snowball sampling. In addition, we conducted eight interviews with other Indigenous leaders or members of local organizations to fill knowledge gaps and to gain a deeper understanding of some of the findings of the interviews with community members. For most of the interviews, we traveled to the communities with a local research assistant who helped us follow cultural protocols. We adhered to key ethical principles including Free, Prior, and Informed Consent (FPIC). Due to weak network coverage, we could not arrange our visits in advance. Upon arrival, we sought the community governor or the highest-ranking leader present and only continued our visit and conducted interviews with their consent. All but two interviews were voice-recorded (with informed consent) and transcribed.^7^ These other two interviews were documented with notes. All information was systematized and coded in NVivo software for analysis.

## Results and discussion

We start the results section with a general observation: at the time of data collection, none of the sampled projects had made a significant positive contribution to the communities’ livelihoods. Poverty was an issue in all the communities we visited, and even though many interviewees were against the project—because these projects went against Indigenous beliefs, or because they were not in agreement with potential land-use changes the projects might cause—many saw these projects as an opportunity to improve their livelihood, even if it was only for very basic needs such as school supplies, soap, or salt. This observation is not surprising because several studies of other REDD+ projects have come to similar conclusions. However, what is striking is that the mere prospect of money already had a profound impact because the communities in our sample had, at the time of interviewing, received either very little or no carbon payments. In the remainder of this section, we discuss the projects’ observed effects on community governance, elite capture, and transparency, and we discuss how these effects contributed to non-violent conflicts such as disputes, internal divisions, contestation, and resistance.^8^

### Local governance effects and elite capture

In general, we discovered that, as a result of the REDD+ projects, community governance is weakened through different mechanisms relating to trust in leadership, governance rights (who makes decisions about the project), and decision-making mechanisms (who was involved in the decision-making). Deciding to participate in the projects was overshadowed by elite capture, which lowered the level of trust between community members and all levels of leadership. In particular, this was because established community decision-making mechanisms were not fully adhered to in some projects. This also contributed to frustrations and disputes between community members and leaders and provided evidence of elite capture in the form of taking control of decision-making, despite governance structures being in place. As a result, trust in leaders was negatively affected and resulted in challenging some leaders’ legitimacy to govern. In all our interviews, we also inquired about the communities’ general decision-making process. In the vast majority, the decisions were made by the leaders, with communities being informed and at times also being asked for their opinion. According to our findings, only when the head of the community signed the contract did it follow the established decision-making process. Also, according to our interviews, it was only when no member of the community contested the project’s legitimacy was the community members’ trust in the community leaders was not affected. Hence, in three of the four investigated projects, established collective decision-making mechanisms were not adhered to, with a small number of elites controlling the decision-making process of a project that influenced land and resource-use policies for the entire collective for several decades. These findings provide further support to existing studies (e.g., Platteau, 2004; Post, 2008; Mansuri & Rao, 2012) that elite capture is less likely to occur in settings with strong governance and strong local institutions.

We found that the contested legitimacy of leaders—as a result of unilateral decision-making—was a source of deep grievances and conflict within local communities. In the Baka Rokarire project (see Table 1), the contract’s legality was contested because the contract was signed by an AATI representative who no longer had the legitimacy to do so (Bermúdez Liévano, 2022).^9^ As a result, the new AATI leadership had to deal with the anger of members who were against the project. In the YUTUCU project, the project’s legality was contested because community members claimed that their constitutional right to prior consultation (*consulta previa*) was violated, resulting in intense conflict with the AATI leadership.^10^ In Colombia, prior consultation is the fundamental right of Indigenous Peoples and other tribal groups to decide on legislative and administrative measures, projects, and other activities within their territories to protect their cultural, social, and economic integrity, and to guarantee their right to participate in decision-making (Amparo Rodríguez, 2008). Despite this, in two previous judicial processes about REDD+ projects, the Colombian courts established that prior consultation was not necessary when projects were initiated by communities. The courts assumed that these kinds of projects fulfilled this requirement (Minambiente, 2020a). However, this decision has been contested by several communities, and recently, the Constitutional Court issued a ruling (Sentencia T-248-24, 2024) that revisited previous judicial decisions relating to the Bakara Rokarire project (Table 1), reaffirming the requirement for prior consultation. This ruling highlighted the lack of clear state guidelines for when prior consultation should take place. It emphasized the need for a protocol to ensure (a) genuine prior consultation, (b) mechanisms to define project ownership, and (c) guidelines for no-harm, economic benefits, and due diligence in the forest carbon market.

As a result of the REDD+ projects, we also saw that disputes arose around power-sharing or around the rules and norms for how communities were organized. In the TICOYA project, unilateral decision-making by the AATI president even contributed to his removal. In this project, the AATI president signed the contract without consulting the heads of communities or the communities themselves.^11^ Two of the three heads of communities interviewed mentioned that they were against the president’s power to sign these contracts.^12^ Despite the fact that the then-president was removed, his unilateral action still generated tension between the community and the AATI leadership. In contrast to the PES program in Chiapas, Mexico—investigated by Corbera et al. (2020)—a successor to the removed leader was elected, but as a result of elite capture in the decision-making around the project, it seems that the old norms of decision-making were challenged and continued to fragment the collective action of resources and territorial governance, as emphasized by the opinion of a member of the Piedra Ñi community (interview (IV) 06):

*…the sale of carbon is an issue, it brings political, organizational or traditional conflict to the territories.*

### Disputes about (anticipated) flows of capital

In addition to the conflicts relating to unilateral decision-making, we found that the very prospect of incoming funds divided the communities, causing mistrust within and between communities but also between communities and the project intermediary. Some community members felt that the AATI leadership was allied with the intermediary and did not represent their interests.^13^ This is a strong finding in light of the fact that one of the foundations of these communities is trust. Community members were also frustrated because some communities had to pay the price for mismanagement and the mistakes of former AATI leaders.^14^ In one interview, a leader also voiced the community’s suspicion that leadership received monetary incentives to sign the contract. This caused frustration, and naturally, it negatively impacted trust in those leaders.^15^ Furthermore, we found that while there was general mistrust about who receives what,^16^ some interviewees also mentioned that this led to conflict within their communities or between the communities.^17^ As a member of the Buenos Aires community said (IV 08):

*…when they talk about money, everyone wants money. They create needs (…). All the communities want to have their [boat] engine, their things, they have many illusions, which build up. The fights start…*

We also found that how (anticipated) money was supposed to be distributed was a polarizing topic and that these preferences were also linked to trust in leaders. While some respondents either had no opinion or the issue was not covered in the interview, others indicated that money should be spent on collective investments.^18^ The majority, however, indicated that funds should either be distributed to families individually or be based on current needs,^19^ which might not be possible due to project conditions. However, according to one community head, distributing money to families individually had led to conflict in the community in the past.^20^ In one project specifically, there also seemed to be an issue with previous fund mismanagement, and this was why community members did not trust some leaders with money and were against funds being channeled through them.^21^ The relationship between perceived trust and the capability of leaders to manage money was also discovered by Pham et al. (2014), although this was more generally related to issues of the organizational capacity of Indigenous organizations (Jiménez, 2007). Once money comes in, these different opinions on how to channel and distribute money could trigger conflict in the communities, but the intensity of the conflict is likely mitigated by the prior internal lack of capacity to manage funds.

Another issue we discovered in relation to the monetary benefits of a project regarded the value proposition of project intermediaries. When entering a collaborative resource-governance system, communities are increasingly challenged about how and what to negotiate with intermediaries in relation to contract terms, and to distinguish legitimate actors from fraudulent actors (Aguilar-Støen, 2017). Intermediaries sometimes propose conditions to communities that are better than the current conditions, and this leads to conflicts between families and leaders of the same AATI.^22^ For instance, the head of the Santa Marta community (IV 15) mentioned that a company had offered them a monthly minimum salary per family (about USD 200), illustrating the associated conflicts like this:

*…there is a company [intermediary], they say that they will pay more than one minimum wage to each family, monthly, so that always generates divisions everywhere.*

In the Vaupés department, it has also been problematic that communities sign with different intermediaries for different deals—some of which were considered more favorable than others. This then led to tensions between those communities.^23^ In their study of REDD+ projects in Costa Rica, Wallbott and Florian-Rivero (2018) also identified this as a factor that caused tension. One intermediary’s communication strategy was also highlighted to be problematic because they talked to heads of communities within the same association individually, causing confusion and problems between communities. Hence, the intermediary negotiated the terms of the collaborative resource-governance system with leaders individually, even though the collective as a whole was affected by the decisions.

### Transparency is an issue at all levels

Our results suggest that lack of information is a major issue, particularly when it comes to the distribution of (prospective) funds. This was mentioned in almost every community as a source of conflict of some sort either between families of the community or between communities themselves. The following quote from an interview with a member of the Timbo Rio community highlights this (IV 01):

*They [community members] want to know sometimes, why that community received more [money], why we received less, they ask “why are you stealing the money?”, that's how they say it. There have always been conflicts and there always will be, as long as there is no collective work.*

Mistrust also seems to be connected to a lack of transparency and access to information more generally. In all projects, there is limited clarity about the projects’ benefits, with several interviewees stating that very little information was being made available to them. This has caused tension between the intermediary and community leadership^24^ but also between the AATI and the community leaders.^25^ This finding supports the evidence found by Montoya-Zumaeta et al. (2021) that the sharing of information is an issue at all levels. One particular issue also seems to be a lack of transparency as to how much the credits are sold for.^26^ This likely results in dissatisfaction with the intermediary. In all of the projects, at least one person mentioned that they were unhappy with the intermediary, either because they were frustrated with little or no money coming in^27^, or that there were irregularities that the intermediary did not explain.^28^ In many communities, people voiced their frustration with the current intermediary and wanted to work with a different company that offered them a more equitable deal.^29^ Many interviewees felt that their benefits fell short, and this contributed to the build-up of frustration and the contestation of the project, thus exacerbating some of the conflicts discussed above. The dissatisfaction with intermediaries was highlighted by a member of the Timbo Rio community (IV 03):

*The fact that we take care [of the forest] and people or companies give us money, that's good. But the execution process has been bad. There are many intermediaries, and projects are not directly between the Indigenous People and the companies [buyers]. There are always some foundations, some corporations that interfere in the process, and in the end, they are the ones who benefit more than we ourselves as Indigenous Peoples. That intermediation, I don't agree with that.*

Accessing information is challenging at every level, and the design of socialization activities is likely related to this issue. Oftentimes, community members knew nothing more than the name of the project, which this quote from a member of the San Martin community shows (IV 33):

*At the moment, within the community there is only the name of the project… People don't know what it means, the only thing they know is that air is bought, but beyond that, people don't know anything.*

The unknown causes conflict and mistrust in the communities, so transparency is crucial to mitigate those conflicts. When we inquired about the respondents’ knowledge about a contract’s conditions, responsibilities, and benefits, we expected that community leaders at least would be aware of them, because it was more likely that this information would be included in the contract negotiations. However, only five of the 16 leaders interviewed (all AATI leaders or heads of communities) had what we would consider a good knowledge of the first two categories. They therefore knew the basic contract conditions—such as duration and/or how benefits from the carbon sales would be split—and they knew about the responsibilities of participation, but they were unclear about how much money would come in or when it would come in. To a certain extent, this is not surprising because estimating the contract value at the start of the project would mean that the intermediary would already know how many credits would be sold and at what price, and this is nearly impossible due to market price fluctuations, currency exchange rates, and unplanned deforestation. However, the heads of communities (or AATI leaders) should know the payment schedule and how benefits are distributed because that way they can manage the financial expectations of community members better. This would then contribute to conflict mitigation. We realized that the design of socialization activities might be part of the transparency issue. In the TICOYA project, a socialization event where the contract conditions were explained was mentioned to us.^30^ However, the event only took place in one community (Naranjales). This made it difficult for community members and some leaders to attend because most of them lacked the time or money to travel to another community for this kind of activity. A similar situation was explained to us in the Makaro project, where one interviewee^31^ told us that they were invited to meetings, but if the meetings did not take place in their communities, they usually did not attend because travel costs were seldom refunded. For maximum reach, socialization activities must take place within the communities. Also, socialization activities often did not seem to meet their goal because community members either still had trouble understanding the projects^32^, or they said that the project was not explained well to them.^33^ We also found that project contracts were not openly shared with all community leaders, not even with the community heads.^34^ However, when we talk about formal contracts, payment schedules, or contract conditions such as benefits and obligations, we are in the world of business. But for Indigenous Peoples, the forest and the territory in general have much more profound meanings, which might explain why Indigenous leaders also know so little about the contracts. The following comment by a leader of the Timbo Rio community emphasizes these different ontologies (IV 03):

*Since I was a child, I was taught not to cut down too many trees, to respect the river because it is also a human being that does us a favor. We were taught that trees give us food, they give us to make houses, they give us to exist, they give us medicine, they give us water. But in the world of money, it is not worth what we are talking about. It is money, it is dollars, it is currency, every day everyone wants to become richer to live better. But in the Indigenous world, it's not that.*

## Conclusion

Despite being challenged as an effective climate-change mitigation instrument (e.g., Guizar-Coutiño et al., 2022; Pelletier et al. 2016; West et al., 2020), and despite a growing body of evidence of negative local social consequences, the forest carbon sector is expanding rapidly and project intermediaries, multilateral organizations, and some conservation NGOs still hold to their narrative of multiple wins of carbon offsetting. Often it is said that projects enable a “triple win,” including continuous economic growth, climate-change mitigation, and positive socioeconomic development outcomes in project countries. Through these processes, carbon has become a tradable good, which together with the urgent need to protect the world’s forests has created a rush to develop forest carbon projects, such as the REDD+ projects. The majority of these projects are implemented in rural areas of the Global South on the lands of ethnic, peasant, or other marginalized communities. These communities are independent social constructs based on heterogeneous norms and institutions. The goal of this study—looking through the lens of community governance, elite capture, and transparency—was to investigate how REDD+ projects implemented on collective territory contribute to non-violent conflicts such as internal divisions, disputes, contestation, or resistance within and between Indigenous communities. While we have focused on Indigenous communities in the Colombian Amazon, our results can inform other contexts with autonomous community governance in places where REDD+ or other land-based carbon interventions are implemented.

Our results are based on interviews conducted within 10 Indigenous communities in the Colombian Amazon that participated in one of four REDD+ projects. In general, we found that the projects have so far failed to make a meaningful contribution to the livelihoods of these communities, whose members, to a great extent, struggle with poverty. Our results even suggest that these projects affect communities negatively. We found that the lack of information-sharing, and possibly also ill-designed socialization activities, contribute to mistrust, frustration, and conflict between and within communities as well as with the project intermediary. This is closely tied to issues related to the distribution of prospective funds. Furthermore, we found a close connection between who decided to participate in the project (and signed the project contract with the intermediary), perceived project legitimacy, and the communities’ trust in its leaders. The only project where no community member contested the project in the interviews was the project where the head of the community—rather than a leader from the community association or one governance level above the community head—decided to participate

This study provides evidence that even when there is clarity about land tenure rights, and when there are systems of self-governance or community governance in place, internal conflicts persist. We also saw internal contestation against formerly established decision-making processes as a result of projects in which some leaders challenged the power of one individual to decide on a project in the name of several communities that could affect land use for several decades. While almost half of the interviewees are in general not against such projects—because of the prospect of some poverty alleviation—all communities voiced discontent about the project intermediary, either because the interviewee considered the project to be a “bad deal,” or because there were discrepancies with the intermediary, which remained unexplained. The perception of a “bad deal” is mostly based on the fact that communities received little to no monetary benefit, and they compared that with what other communities get.

Our study contributes to the understanding of the complex interplay between trust, governance, and community participation, all of which are crucial structural elements that influence the likelihood of conflicts or governance breakdowns in REDD+ projects. We found that elite capture significantly undermines trust within communities, leading to internal divisions and a lack of cohesion. This, in turn, weakens the established governance structures because decision-making becomes concentrated in a few hands, often sidelining broader community involvement.

Moreover, our findings underscore that low levels of participation and engagement across the entire community are key factors that lead to the disruption of governance. Projects in which community members felt excluded from initial decision-making, or in which there was a lack of information about the projects’ goals and benefits, were more prone to internal conflicts. This highlights the importance of ensuring robust mechanisms for broad community involvement, where decision-making is both transparent and participatory, in line with the principles of prior consultation. Our case studies reaffirm the Constitutional Court’s stance on the need for prior consultation, emphasizing that comprehensive community engagement can mitigate the risks of governance fractures and elite capture.

Also, to increase the legitimacy of these projects’ within communities, project intermediaries should aim to include the heads of communities in contract negotiations. Including them would improve the flow of information to the communities and would leave less room for elite capture. However, this would have to happen in such a way that autonomous governance structures are respected. Furthermore, better expectation management at the community level could mitigate some of the perceptions that the project is a “bad deal,” which presupposes more extensive transparency at all levels. While we are not arguing that project intermediaries have bad intentions, policy-makers—such as those who develop carbon standards or project registries—should publish more information related to the projects, including contract conditions, as more transparency in the sector would be beneficial to the community. While information-sharing and transparency can be crucial for more inclusive project design, it also comes with a few practical challenges. At the local level, remote communities are hard to reach, and this hinders regular communication between AATI and community leadership. At the project level, effective information-sharing might be hindered through both cultural and language barriers. The worldview between project developers—who are driven by the market value of forests—is vastly different from Indigenous cosmology. Implementing institutional measures to increase information-sharing, not only at the project level but also about Indigenous knowledge systems, could contribute to a greater balance of power and more balanced negotiations between diverse systems of thought and ontologies around a common goal.

Finally, we provide evidence that the likelihood of negative impacts from REDD+ projects is closely mediated by the levels of community governance and participation. Strengthening these mechanisms can foster trust, ensure more equitable decision-making, and enhance the resilience of governance structures. This suggests that policies that promote prior consultation and transparency are not only a legal necessity but also a practical tool for reducing the unintended consequences of climate-change mitigation initiatives in Indigenous territories.

## Figures and Tables

**Figure 1.**
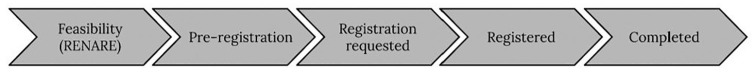
Steps of project registration process (simplified).

**Figure 2.**
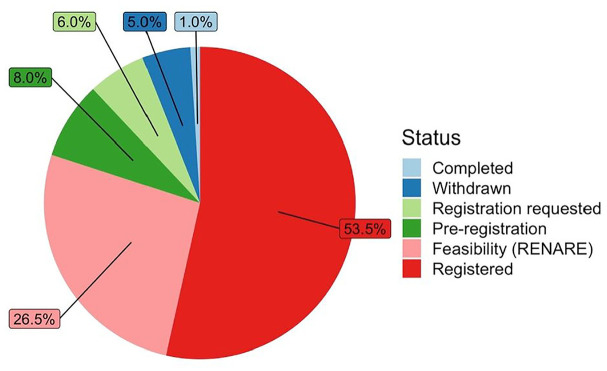
Distribution of all projects by status (2022).

**Figure 3.**
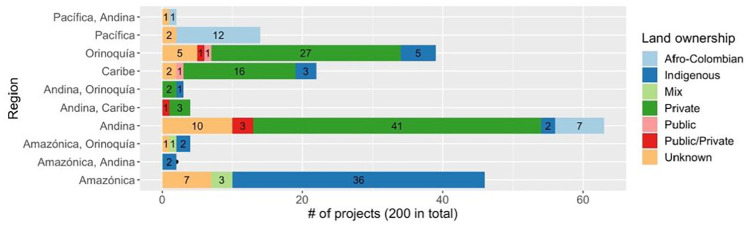
Projects by region and land ownership.

**Figure 4.**
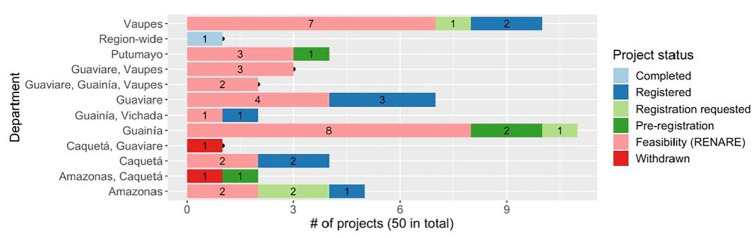
Amazon region: projects by department and status.

## Appendices

### Appendix 1: Case selection process

#### Colombian forest carbon landscape

Before making our case selection, we had to get a complete picture of the Colombian forest carbon landscape including information on the number and location of projects, status of activities, and land ownership. In Colombia, a national database (RENARE) on carbon projects exists.^35^ Despite the fact that RENARE was created in part to increase transparency, the database offers very little information on the projects. Using secondary materials such as project design documents (PDD), we complemented the information in RENARE to get better insight into the Colombian forest carbon project landscape. On 15 June 2022, 200 REDD+ and other forest carbon projects were listed with RENARE. Over 50% of these projects were registered and 1% was completed, which means that the crediting period has ended. In six percent of the projects, the intermediary had requested registration and about 35% of the projects were in the early stages of development (stati = feasibility (RENARE) and pre-registration). Five percent of the projects in the database were withdrawn, which can happen at any stage of the project development process. Figure 1 gives a simplified overview of the development steps of a carbon project and Figure 2 shows the distribution of all projects by status as of 2022.

#### Case selection process

Geographically, we limited our study to the Amazon region because it has the highest concentration of projects on Indigenous reserves (Figure 3). Then our case selection was driven by (a) project status and (b) security. The first criterion was crucial because to fulfill the research aim we had to focus on communities that have been participating in a project for a while. This is because we wanted to allow for sufficient time for socialization activities to take place. Socialization activities are conducted by the project developer and sometimes delegated community members and aim to inform communities about the projects and to answer any questions. We decided to focus on registered projects or projects requesting registration. This requirement is not met by projects in Putumayo (Figure 4). We also excluded the project in Guainía (Flor de Inírida) because it is one of the most contested REDD+ projects in Colombia. Socialization activities started in 2011 but the project’s actual implementation status remains unclear and one of our interviewees stated that no activities are being implemented.^36^ Because of this uncertainty, we decided to exclude it. The general security situation was rather fragile in Caquetá and Guaviare during the time of our fieldwork so we excluded projects there.^37^

In the Vaupés department, three projects meet the project status criterion, all within the Great Vaupés Indigenous Reserve (*Gran Resguardo de Vaupés*). We included all three projects in our study. In the Amazon department, we excluded two projects because they are located within the Predio Putumayo Reserve, which was difficult to get to logistically. This selection criteria resulted in four selected projects for the study, three in the Vaupés department and one in the Amazon department as described in Table 1, section “ Local institutions’ vulnerability to elite capture” of this article.

## Appendix 2

**Table:** List of interviews.

Project	Interviewees (ID, community, AATI, status)
1 Makaro	# 1: Timbo Rio (ATICAM), non-leader # 2: Timbo Rio (ATICAM), non-leader # 3: Timbo Rio (ATICAM), captain (head of community) # 4: Timbo Rio (ATICAM), non-leader # 5: Timbo Rio (ATICAM), leader
2 Baka Rokarire	# 6: Piedra ñi (ACAIPI), AATI president # 7: Piedra ñi (ACAIPI), AATI leader
3 YUTUCU	# 11: Mituseño-Urania (AATIAM), non-leader # 12: Mituseño-Urania (AATIAM), captain (head of community) # 13: Mituseño-Urania (AATIAM), non-leader # 14: Mituseño-Urania (AATIAM), non-leader # 15: Santa Marta (ASOUDIC), captain (head of community) # 16: Santa Marta (ASOUDIC), non-leader # 17: Santa Marta (ASOUDIC), non-leader # 18: Santa Rosalia (ASATRAIYUVA), non-leader # 19: Santa Rosalia (ASATRAIYUVA), former leader (other than captain) # 20: Santa Rosalia (ASATRAIYUVA), vice-captain # 21: Santa Rosalia (ASATRAIYUVA), captain (head of community) # 22: Bocas de Yí (ASATRAIYUVA), leader (other than captain) # 23: Bocas de Yí (ASATRAIYUVA), non-leader
4 TICOYA	# 28: Valencia, (ATICOYA), president ATICOYA # 29: Puerto Esperanza (ATICOYA), curaca *(head of community)* # 30: Puerto Esperanza (ATICOYA), non-leader # 31: San Martin (ATICOYA), non-leader # 32: Puerto Esperanza (ATICOYA), non-leader # 33: San Martin (ATICOYA), vice-curaca # 34: San Martin (ATICOYA), non-leader # 35: San Martin (ATICOYA), curaca *(head of community)* # 36: San Francisco (ATICOYA), curaca *(head of community)* # 37: San Francisco (ATICOYA), AATI leader
Other interviews	# -1: Former employee of a nature conservation organization active in multiple Latin American countries # 00: Former leader of OPIAC # 08: Buenos Aires (ACTIVA), AATI leader # 09: Employee of a local foundation that focuses on Indigenous Peoples’ rights and well-being and leader of the AATI “ATIQ” (joint interview) # 10: Buenos Aires (ACTIVA), AATI leader # 24: Leader AATI “ACTIVAM” # 25: Employee of a local foundation that focuses on Indigenous Peoples’ rights and well-being
Total	38 interviewees (37 interviews)

## Notes

1. Reducing Emissions from Deforestation and Forest Degradation.

2. United Nations Framework Convention on Climate Change.

3. Intermediaries can be for-profit entities or non-profit NGOs. Albeit, for-profits are more common than non-profit entities.

4. Masbosques is a Colombian non-profit organization.

5. The YUTUCU project was withdrawn from the registration process in October 2022 out of frustration with the duration of the process (AATIAM et al., 2022). Despite the fact that activities were implemented, no payments were made because of a lack of certification.

6. South Pole Group is a globally operating company headquartered in Switzerland.

7. This study was approved by the Ethical Review Board of Utrecht University (ID: Geo S-22748)

8. We did not treat our interviewees as a homogeneous group because we wanted to reflect their individual opinions. For this reason, we refer in the footnotes to interview IDs and the interview community (Appendix 2). The quotes were translated from Spanish by the authors.

9. Also mentioned in institutional interview 25.

10. Member of the Santa Marta community (15).

11. Members of communities Valencia (28), Puerto Esperanza (29), and San Francisco (36, 37).

12. Members of communities San Martin (35) and San Francisco (36).

13. Members of communities Puerto Esperanza (30, 32).

14. Members of communities Valencia (28), Puerto Esperanza (29, 30), and San Francisco (36, 37).

15. While a similar comment was made in one of our institutional interviews, this allegation could not be independently verified.

16. Members of communities Timbo Rio (1), Mituseño-Urania (13), San Martin (33), and San Francisco (36).

17. Members of communities Timbo Rio (1), Mituseño-Urania (13), and San Francisco (36).

18. Members of communities Timbo Rio (02), Piedra Ñi (06), Santa Marta (15), and San Martin (35).

19. Members of communities Timbo Rio (01, 04, 05), Piedra Ñi (07), Mituseño Urania (12, 13), Santa Rosalia (18, 19, 20, 21), Bocas de Yì (22), Valencia (28), Puerto Esperanza (29), and San Martin (33,34).

20. Member of the Santa Marta community (15).

21. Members of communities Mituseño-Urania (14), Santa Marta (15), Santa Rosalia (18), and Bocas de Yí (23).

22. Members of communities Santa Marta (15), Santa Rosalia (19), and Bocas de Yí (23).

23. Member of the Timbo Rio community (3).

24. Members of communities Timbo Rio (03), Puerto Esperanza (29), San Martin (35), and San Francisco (36).

25. Members of communities Puerto Esperanza (29, 30), San Martin (34), and San Francisco (36).

26. Members of communities Timbo Rio (03), Puerto Esperanza (29, 30), San Martin (35), and San Francisco (36).

27. Members of communities Valencia (28), Puerto Esperanza (29), San Martin (34), and San Francisco (36).

28. Member of Timbo Rio community (03).

29. Members of Timbo Rio community (03), Santa Rosalia (21), Valencia (28), Puerto Esperanza (29), San Martin (35), and San Francisco (36).

30. Member of the San Francisco community (37).

31. Member of the Timbo Rio community (2).

32. Members of communities Bocas de Yí (22) and San Martin (34).

33. Members of communities Santa Rosalia (19) and Bocas de Yí (23).

34. Members of communities Mituseño-Urania (12), Bocas de Yí (22), and San Martin (34).

35. RENARE has been offline since August 2022.

36. Interview with a former employee of a regional nature conservation organization (interview ID-1).

37. We recognize that REDD+ projects are embedded in broader socio-political contexts and the particular case of Colombia in the context of the civil conflict. While this may contribute to the problems of power asymmetries discovered in this study, understanding the context of the conflict on REDD+ projects was not part of the central research question of this study.
